# I fear you’re getting too close: neural correlates of personal space violation in paranoia

**DOI:** 10.1038/s41537-025-00625-x

**Published:** 2025-05-21

**Authors:** Mélodie Derome, Frauke Conring, Nicole Gangl, Adamantini Hatzipanayioti, Florian Wüthrich, Maximilian Rüter, Stephanie Lefebvre, Sebastian Walther, Katharina Stegmayer

**Affiliations:** 1 Translational Research Center, University Hospital of Psychiatry and Psychotherapy, University of Bern, Bern, Switzerland; 2 Graduate School for Health Sciences, University of Bern, Bern, Switzerland; 3 Translational Imaging Center (TIC), Swiss Institute for Translational and Entrepreneurial Medicine, Bern, Switzerland; 4 Department of Psychiatry, Psychosomatics, and Psychotherapy, Center for Mental Health, University of Würzburg, Würzburg, Germany

**Keywords:** Schizophrenia, Biomarkers

## Abstract

Increased personal space (PS) is a clinically relevant marker for paranoia. Neuroimaging evidence suggested limbic and prefrontal circuit alterations related to threat processing and emotion regulation (i.e., amygdala, fronto-parietal cortex). We hypothesize that patients with paranoia will respond with altered activation in PS-relevant brain areas (i.e., limbic regions, fronto-parietal cortex) toward personal space intrusion. We included 79 participants with various degrees of paranoia severity; 49 patients diagnosed with schizophrenia and 30 controls. In this fMRI study, participants passively viewed pictures of facial expressions in approaching, static, or retracting motions. Violation of PS was modelled with the approaching faces condition. We used firstly a cut off to separate patients in high and low paranoia, and secondly the continuous variations of paranoia severity to understand the full picture. While participants were passively watching faces approaching them in contrast to static faces, group comparison revealed that patients with high paranoia showed hypoactivity mainly in the OFC when compared to patients with low paranoia, and hypoactivity in dlPFC and dPCC when compared to controls. Further, paranoia severity was positively associated with activation of the right hippocampus. Altered neural activity in the OFC, dlPFC, and hippocampus may well reflect the neural responses to the paranoid experience of threat and provide evidence for the hypothesized association between limbic dysfunction and paranoid threat. Modelling of paranoia severity captures variance in neural response to approaching threat, which may be previously undetected due to heterogeneity when examined at the group level.

## Introduction

The positive symptoms of schizophrenia include symptoms related to the paranoid experience of threat (i.e., paranoid delusions, hallucinations, hostility, or suspiciousness) as well as symptoms with positive emotional valence (i.e., grandiosity or paranoid experience of power^1^). Paranoia includes a number of distressing thoughts such as mistrust, suspiciousness, persecutory beliefs and, at the extreme end of the spectrum, delusions^2^. Paranoid beliefs are the most commonly reported delusion among individuals diagnosed with schizophrenia^3^, although 10-15% of the general population also experience paranoid thoughts, suggesting that paranoia exists on a continuum across both subclinical and clinical levels^4^. A large body of evidence has shown alterations of the limbic system (amygdala, hippocampus), medial frontal cortex, and cingulate cortex linked to paranoid experience of threat and persecutory delusions in schizophrenia patients^5–12^. Paranoia manifests similarly in subclinical and clinical populations, suggesting that they share neural mechanisms^13–15^, therefore it is of importance to consider paranoia both with a categorical and a continuous approach.

Personal space (PS) represents the area around the body that individuals maintain between themselves and others during social interactions^16^, reflecting nonverbal social behaviors such as intimacy between people^17^. Violation of one’s PS triggers feelings of discomfort, stress, and anxiety^18^, urging to reinstate safety and appropriate distance by moving farther away^19,20^. Enlarged and inflexible PS have been consistently reported in patients with schizophrenia^21–27^, and were not only associated with negative^25–27^ but also with positive symptoms^28^. PS measures detected a subgroup of patients with positive symptoms; those currently experiencing paranoid threat^28,29^, suggesting that PS regulation in schizophrenia is underlined by distinct contributions of positive symptoms^28^. To behaviorally simulate the intrusion into one’s PS, previous studies have used the reliable measure of the *stop distance paradigm*, in which participants indicate the minimal tolerable distance to an approaching experimenter^20,28^. In the present fMRI study, we simulated intrusion into one’s PS while participants were lying in the scanner by means of videos of facial expressions enlarged (zoomed-in) to make the person appear very close, almost touching. The neural correlates of the approaching faces fMRI task have already been investigated in healthy individuals^30,31^, in borderline personality disorder^32^, in violent offenders^33,34^, in relation to anxious attachment^35^, and in patients with schizophrenia^36^. However, no study to date has investigated the neural correlates of the approaching faces paradigm as a function of paranoia severity in schizophrenia patients. Both studies from Holt and colleagues and findings from Vieira and colleagues suggested the involvement of a parietal-frontal network in healthy controls and schizophrenia patients while watching approaching faces (intraparietal sulcus and ventral premotor cortex)^30,31,36^. In addition, a series of animal and human studies have shown that PS representation is mediated by neurons in the parietal cortex, the putamen, and the premotor cortex^37,38^.

Paranoia and impaired PS may share some neuroimaging markers in the limbic system, specifically the amygdala and hippocampus. However, evidence on neural correlates of PS regulation in paranoia is still missing. To address this need, the objective of the present study is to explore paranoia symptoms, both categorically (high vs low paranoia vs controls) and continuously (paranoia severity) in relation to the neural underpinnings of threat processing in schizophrenia patients and matched healthy controls. Personal space violation is modeled in the present study as the approaching faces condition in comparison to the static condition (the main contrast of interest is Approach > Static). Given that limbic and PFC circuit pathology relate to threat processing and emotion regulation, we hypothesized that patients with higher levels of paranoia will respond with altered activation in PS-relevant brain areas (i.e., amygdala, hippocampus, PFC, cingulate cortex) toward personal space intrusion. The continuous approach might yield similar neural correlates, we hypothesize that symptom heterogeneity may be lost in the categorical analysis that the continuous approach could capture, thus combining both approaches will allow us to appreciate the whole picture. Altogether, our theoretical model suggests that limbic and affective circuit dysfunction drives paranoia, which in turn impacts how individuals respond to social threat.

## Results

### Clinical measures

Participants demographics are reported in Table 1. Patients with schizophrenia and controls did not differ on age, sex, and intracranial volume. But they differed in GPTS-A and GPTS-B mean scores as anticipated.

**Table 1:** Demographic information of controls and patients’ groups.

	Controls	Patients	Statistics	Effect sizes (Cohen’s d)
*n*	30	49	–	–
DSM-V criteria	– –	Schizophrenia (*n* = 28), Schizoaffective disorder (*n* = 9), Schizophreniform disorder (*n* = 12)	–	–
Age	34.8(12.1)	37.6 (13.7)	*t*(77) = −0.935, *p* = 0.352	*d*=−0.217
Sex	Males: 18(60%) Females: 12(40%)	Males: 30 (61.22%) Females: 19 (38.78%)	*χ*^*2*^ = 0.017, *p* = 0.914	–
OLZ-24 h	–	15.2 (10.3)	–	–
GPTS-A	19.7(3.36)	36.2 (16.1)	*t*(77) = −5.51, *p* < 0.001	*d* = −1.28
GPTS-B	17.1(2.20)	33.3 (19.1)	*t*(77) = −4.61, *p* < 0.001	*d* = −1.07
PANSS total	–	67.3 (15.2)	–	–
PANSS positive	–	17.5 (5.62)	–	–
PANSS Negative	–	16.4 (5.90)	–	–
TIV	1522(197)	1426 (168)	*F*(2) = 2.33, *p* = 0.107	*d* = −0.559
Duration of illness	–	8.23 (7.28)	–	–

	Controls	Patients with paranoia	Patients without paranoia	Statistics	Effect sizes
*n*	30	18	31	–	–
Age	34.8 (12.1)	42 (13.3)	35.1 (13.5)	*F*(2) = 2.09, *p* = 0.131	η²=0.052
Sex	Males: 18 (60%) Females: 12 (40%)	Males: 10 (55.55%) Females: 8 (44.44%)	Males: 20 (64.52%) Females: 11 (35.48%)	*χ*^*2*^ = 0.395, *p* = 0.821	–
OLZ-24 h	–	19.8 (11.3)	11.8 (8.05)	*t*(40) = 2.68, *p* < 0.011	*d* = 0.837
GPTS-A	19.7 (3.36)	44.9 (14.5)	31.1 (28.0)	*F(2)* = 25.6, *p* < 0.001	η²=0.403
GPTS-B	17.1 (2.20)	56.7 (13.7)	20.5 (5.46)	*F(2)* = 180, *p* < 0.001	η²=0.825
PANSS total	–	73.6 (13.7)	63.8 (15.1)	*t(46)* = *2.21, p* = *0.032*	*d* = 0.666
PANSS positive	–	21.4 (5.62)	15.4 (4.45)	*t(46)* = *4.02, p* < *0.001*	*d* = 1.21
PANSS Negative	–	15.6 (5.26)	16.8 (6.27)	*t(46)* = *-0.680, p* = *0.500*	*d* = −0.205
TIV	1522 (197)	1426 (168)	1510 (138)	*F*(2;51.4) = 2.33, *p* = 0.107	*d* = −0.559
Duration of illness	–	12.5 (8.37)	5.87 (5.00)	*t*(46) = 3.34, *p* = 0.002	*d* = 1.01

GPTS stands for Green et al. Paranoia scale (A for ideas of reference, B for ideas of persecution), TIV for Total Intracranial Volume. OLZ_24 h represent the olanzapine equivalent. PANSS represents the Positive and Negative Syndrome Scale.

The two groups of patients (SZ-high-paranoia and SZ-low-paranoia) further differed in medication, duration of illness, and PANSS total and positive.

### Primary fMRI analyses—categorical approach

Patients with high paranoia (SZ-high-paranoia) showed hypoactivity lateralized on the right hemisphere during the approaching (vs static) condition when compared to patients with low paranoia (SZ-low-paranoia) in the orbitofrontal cortex (OFC), cerebellum, occipital, superior temporal gyrus, and inferior parietal lobule. When controlling for medication dose, patients with high paranoia also exhibited hypoactivity in the r-OFC compared to low-paranoia patients, indicating that the overall effects observed persist even after accounting for medication differences.

**Fig. 1:**
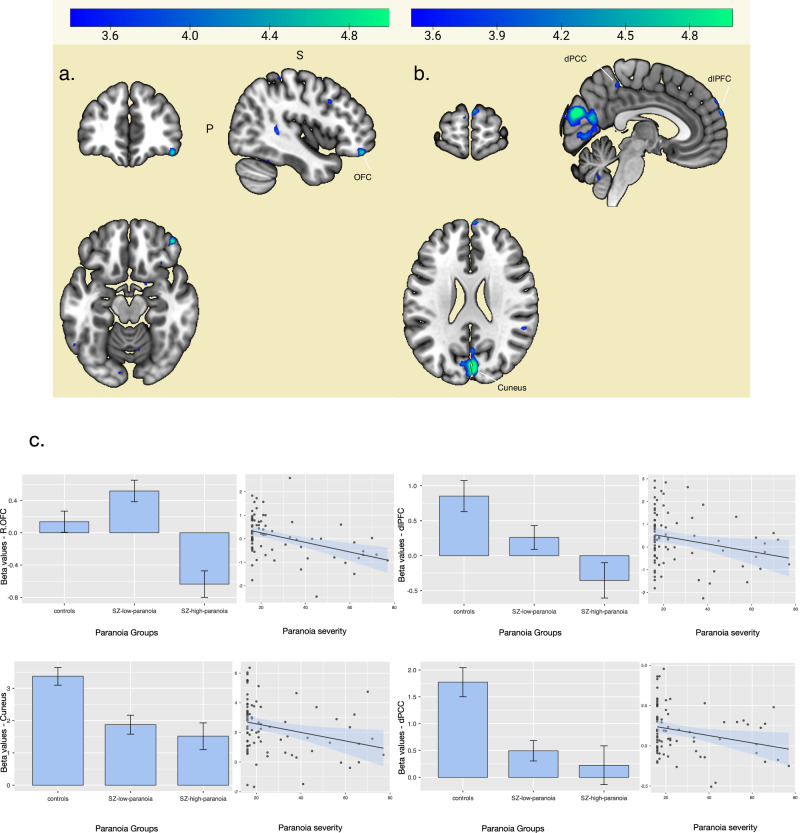
Comparison between groups during interpersonal infringement (Approach > Static). **A** SZ-low-paranoia>SZ-high-paranoia; **B** Controls > SZ-high-paranoia note. **A** Patients with high paranoia showed hypoactivity of the R-orbito frontal cortex (in Blue) compared to low paranoia. **B** Patients with high paranoia showed decreased activity in cuneus, dorsal posterior cingulate (dPCC), and dorso-lateral prefrontal cortex (dlPFC) (in Blue) compared to controls. **C**. For illustration purposes, we extracted beta values from each region that showed reduced activity (from A and B contrasts) and computed correlations with paranoia severity (GPTS-B as a continuous variable. Error bars represent standard deviation (SD).

When compared to controls, SZ-high-paranoia exhibited decreased activity in bilateral dorsolateral prefrontal cortex (dlPFC), bilateral dorsal posterior cingulate cortex (dPCC), as well as parietal (superior parietal lobule), temporal (superior and middle temporal gyrus), and occipital regions (fusiform, cuneus). See Fig. 1, Table 2.

**Table 2:** fMRI comparison between groups based on the GPTS-B cut off during interpersonal space infrigement (Approach > Static).

A. SZ-high-paranoia < SZ-low-paranoia								
Cluster p(FWE-corr)	Cluster p(FDR-corr)	Cluster number of voxels	Peak p(FWE-corr)	Peak p(FDR-corr)	Peak T values	Coordinates (x y z) in mm	Hemisphere and lobes	Region details
0.018	0.008	66	0.184	0.601	5.29	44 44 −16	Right frontal lobe	Orbito frontal gyrus, BA47
			1	0.899	4.09	34 38 −20	Right frontal lobe	Orbito frontal gyrus, BA47
			1	0.899	3.94	46 50 −8	Right frontal lobe	Anterior PFC, BA10
0.033	0.012	59	0.724	0.67	4.78	10 −66 −28	Right cerebellum	posterior lobe, uvula
			0.81	0.67	4.7	10 −64 −20	Right cerebellum	posterior lobe, declive
			1	0.985	3.33	4 −68 −12	Right cerebellum	posterior lobe, declive
0.001	0.001	391	0.802	0.67	4.71	4 −80 24	Right occipital lobe	cuneus, BA18
			0.893	0.67	4.61	2 −66 18	Right occipital lobe	Dorsal PCC, BA31
			0.971	0.806	4.46	16 −60 4	Right occipital lobe	lingual gyrus, BA18
0.018	0.008	66	0.91	0.67	4.58	52 −42 16	Right temporal lobe	superior temporal gyrus, BA22
			1	0.915	3.57	56 −42 8	Right temporal lobe	superior temporal gyrus, BA22
0.003	0.003	88	1	0.899	4.07	48 12 36	Right frontal lobe	Middle frontal gyrus, BA44
			1	0.899	3.84	36 12 32	Right frontal lobe	precentral gyrus, BA44
0.051	0.016	54	1	0.899	3.8	42 -38 58	Right parietal lobe	inferior parietal lobule, BA1
			1	0.971	3.39	36 −42 52	Right parietal lobe	inferior parietal lobule, BA7

B. SZ-high-paranoia < Controls								
Cluster p(FWE-corr)	Cluster p(FDR-corr)	Cluster number of voxels	Peak p(FWE-corr)	Peak p(FDR-corr)	Peak T values	Coordinates (x y z) in mm	Hemisphere and lobe	Region details
0.001	0.001	3024	0.001	0.004	6.78	4 −80 24	Right occipital lobe	cuneus, BA18
			0.171	0.237	5.31	2 −66 18	Right occipital lobe	dorsal PCC, BA 31
			0.208	0.237	5.26	−14 −78 10	Left occipital lobe	calcarine cortex, BA17
0.001	0.001	214	0.042	0.127	5.66	12 −42 48	Right parietal lobe	dorsal PCC, BA31
			1	0.764	3.61	0 −44 46	Left parietal lobe	dorsal PCC, BA31
0.001	0.001	155	0.388	0.314	5.08	54 −40 16	Right temporal lobe	superior temporal gyrus, BA22
			1	0.626	3.89	62 −42 16	Right temporal lobe	superior temporal gyrus, BA22
			1	0.861	3.41	60 −48 8	Right temporal lobe	middle temporal gyrus, BA22
0.001	0.001	267	0.67	0.354	4.83	40 −46 −26	Right temporal lobe	fusiform gyrus, BA37
			0.942	0.483	4.53	56 −50 −22	Right occipital lobe	fusiform gyrus, BA37
			0.989	0.483	4.38	46 −42 −18	Right temporal lobe	fusiform gyrus, BA37
0.001	0.001	161	0.691	0.354	4.81	6 64 34	Right frontal lobe	superior frontal gyrus, dlPFC, BA9
			0.961	0.483	4.49	4 62 26	Right frontal lobe	superior frontal gyrus, anterior PFC, BA10
			1	0.512	4.13	−6 64 18	Left frontal lobe	superior frontal gyrus, anterior PFC, BA10
0.001	0.001	242	0.986	0.483	4.4	42 −40 52	Right parietal lobe	superior parietal lobule, BA40
			0.999	0.507	4.22	28 −52 54	Right parietal lobe	superior parietal lobule, BA7
			1	0.94	3.29	36 −38 46	Right parietal lobe	superior parietal lobule, BA40
0.001	0.001	101	1	0.512	4.15	−8 −34 74	Left parietal lobe	post central gyrus, BA1
			1	0.512	4.11	8 −32 78	Right parietal lobe	post central gyrus, BA4
			1	0.691	3.72	−8 −40 60	Left parietal lobe	precuneus, BA31

The contrasts SZ-High-paranoia > SZ-low-paranoia and SZ-high-paranoia > Controls did not yield significant results. Results of the other task conditions comparisons are presented in the supplementary material.

### Primary fMRI analyses—continuous approach

**Fig. 2:**
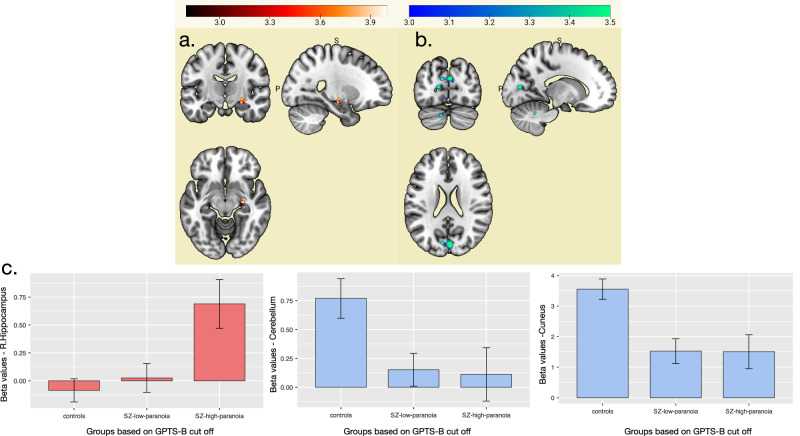
fMRI results of multiple regression between paranoia severity and task conditions during the Approach condition in comparison to the Static condition (Approach > Static) note. Upper part. **A** Paranoia severity was associated with hyperactivity of the R-hippocampus (in Red) during the approaching compared to static condition. **B** and with decreased activity in occipital and cerebellum (in Blue). For illustration purposes. Bottom part (**C**). In Red, Hippocampus beta values extracted from (**A**) the contrast Approach > Static (positive correlation between GPTS-B and paranoia severity) and plotted between groups based on the GPTS-B cut off score ( > 35 group SZ-high paranoia, <35 group SZ-low-paranoia). Post hoc comparisons revealed significant differences for the comparisons controls<SZ-high-paranoia (*t*(75) = −3.781, *p* < 0.001) and SZ-high-paranoia>SZ-low-paranoia (*t*(75) = 3.237, *p* = 0.005). In Blue, Cerebellum and Cuneus beta values extracted from (**B**). The contrast Approach>Static (negative correlation between GPTS-B and paranoia severity) showed significant differences between the groups based on GPTS-B cut off score of 35. Post hoc comparison showed for the cerebellum: controls>SZ-high-paranoia (*t*(77 = 2.470, *p* = 0.041)) and controls > SZ-low-paranoia (*t*(77) = 2.705, *p* = 0.023)), and for the cuneus: controls > SZ-high-paranoia (*t*(77 = 3.240, *p* = 0.005)) and controls>SZ-low-paranoia (*t*(77) = 2.3.774, *p* = 0.001)). Error bars represent standard deviation (SD).

When comparing approaches to static conditions, paranoia severity (as measured with the GPTS-B) was positively correlated with activation in the right hippocampus (coordinates: 28 −16 −10). Additionally, paranoia severity was negatively correlated with activation of regions of the right and left occipital lobes (encompassing the cuneus) and cerebellum. See Table 3, Fig. 2.

**Table 3:** fMRI - Multiple regressions between paranoia severity and task conditions.

	Cluster p(FWE-corr)	Cluster p(FDR-corr)	Cluster number of voxels	Peak p(FWE-corr)	Peak p(FDR-corr)	Peak T values	Coordinates (x y z) in mm	Hemisphere and lobes	Region details
Baseline (Static Neutral)									
Positive correlation GPTS-B*Baseline									
	0.006	0.008	80	0.078	0.173	5.5	−32 10 42	Left Frontal lobe	middle frontal gyrus, BA6
				0.809	0.51	4.69	−30 14 52	Left Frontal lobe	middle frontal gyrus, BA6
	0.163	0.044	42	0.669	0.426	4.82	28 34 18	Right Frontal lobe	middle frontal gyrus, anterior PFC, BA10
	0.025	0.011	63	0.911	0.542	4.57	−22 −83 2	Left Occipital lobe	inferior occipital gyrus, BA18
				1	0.84	4.57	−28 −76 0	Left Occipital lobe	fusiform gyrus, BA18
	0.036	0.011	59	0.993	0.749	4.34	−10 −2 36	Left	ventral anterior cingulate, BA24
	0.027	0.011	62	1	0.84	4.02	−14 −86 −36	Left cerebellum	posterior lobe, Pyramis
Approach > Static									
Positive correlation GPTS-B*Approach>Static									
	0.11	0.028	46	0.001	0.769	4.74	28 −16 −10	Right Limbic	hippocampus
Negative correlation GPTS-B*Approach>Static									
	0.005	0.008	81	0.761	0.975	4.74	4 −80 24	Right Occipital lobe	cuneus, BA18
				1	0.975	3.29	−2 −82 12	Left Occipital lobe	cuneus, BA17
				0.876	0.975	4.62	−2 −60 −48	Cerebellum	Posterior lobe, Pyramis
Approach > Retreat									
Negative correlations GPTS-B*Approach>Retreat									
	0.033	0.016	62	0.745	0.827	4.74	−10 −24 14	Left Limbic	thalamus
				0.939	0.873	4.51	−22 −32 12	Left Limbic	thalamus
	0.002	0.002	97	0.998	0.93	4.24	34 −56 −22	Right Occipital lobe	fusiform gyrus, BA37
				0.999	0.93	4.23	34 −46 −26	Right Occipital lobe	fusiform gyrus, BA37
				1	0.93	3.88	28 −38 −28	Right Occipital lobe	fusiform gyrus, BA37
	0.001	0.001	134	1	0.93	4.12	26 −82 8	Right Occipital lobe	superior occipital gyrus, BA18
				1	0.93	3.99	34 −82 10	Right Occipital lobe	middle occipital, BA19
				1	0.93	3.74	38 −82 0	Right Occipital lobe	inferior occipital gyrus, BA18
	0.036	0.016	61	1	0.93	4	18 −66 −52	Right Cerebellum	posterior lobe, inferior semi-lunar lobule
				1	0.93	3.98	36 −66 −46	Right Cerebellum	posterior lobe, inferior semi-lunar lobule
				1	0.93	3.71	28 −72 −48	Right Cerebellum	posterior lobe, inferior semi-lunar lobule

No voxels survived threshold for the contrasts Retreat > Static, Angry > Neutral, Approach Angry > Approach Neutral. Results of the contrast Approach Angry > Static Neutral are presented in the supplementary material.

Other comparisons between conditions are presented in Table 3 and in the supplementary material.

### Secondary fMRI analyses—results regarding emotional valence analyses

The Angry > Neutral contrast did not reveal significant differences between patient groups. The only observed group-level difference was that patients exhibited hypoactivity in the cerebellum and middle frontal gyrus compared to controls. See supplementary material 2.2.e.

For the regression analyses, none of the tested contrasts, including Retreat > Static, Angry > Neutral, Approach Angry > Approach Neutral, and Approach Angry > Static Angry, yielded significant results. However, an exploratory analysis of the Approach Angry > Static Neutral contrast (without multiple comparison correction) identified the right hippocampus, which was also observed in the primary contrast of interest Approach > Static. See supplementary material 2.3.d.

## Discussion

This study reports the neural correlates of the fMRI approach faces condition in relation to current paranoid threat. We used two approaches to define paranoia symptoms; a categorical approach separating patients in high and low paranoia based on the GPTS-B cut off 35, and a continuum approach assessing paranoia severity continuously among controls and patients.

During personal space violation, as in faces approaching, patients with high paranoia showed hypoactivity mainly in the OFC when compared to patients with low paranoia, and hypoactivity in dlPFC and dorsal posterior cingulate cortex when compared to controls. Further, the continuum of paranoia severity was associated with increased activity of the right hippocampus. Thus, altered neural activity in the affective processing and limbic regions during the approaching faces task, may well reflect the neural responses to the paranoid experience of threat. The categorical and continuous approach yielded different neural correlates of paranoia, helping us to draw the whole picture of the mechanistic model of paranoia.

### Decrease activity in OFC, dlPFC and dorsal posterior cingulate cortex during personal space violation

Generally, cognitive processing in the presence of emotional stimuli has been shown to engage ventral PFC, including the inferior frontal gyrus, medial PFC and OFC^39^. Notably, cognitive control of anxiety states, threat-related distractors, and reappraisal of threat stimuli were associated with lateral PFC and OFC activation^40,41^. Furthermore, in a previous study, Walther and colleagues observed increased connectivity between orbitofrontal and medial PFC as a function of paranoia severity^11^. Our results suggest aberrant activity within orbitofrontal/PFC circuit that reflects threat processing and impaired emotional regulation. Perry and colleagues also observed that patients with orbitofrontal damages exhibited abnormal personal distance preferences (tolerating very short PS distances) compared to healthy controls. In contrast, the only previous study assessing the neural correlates of approaching faces fMRI task in schizophrenia patients found no association with the OFC, yet they did not assess paranoia specifically^36^. The OFC is also known to integrate sensory and social information to guide adaptive decision-making and modulate responses to perceived threats. Recent research^42^ highlights the OFC’s critical function in adjusting expectations based on new information, which is especially relevant in dynamic interpersonal contexts where social cues and perceived threats must be continuously evaluated. In individuals with paranoia, hypoactivation of the OFC may contribute to rigid threat beliefs, impairing the ability to reassess social approach behaviors accurately. This dysfunction could explain why neutral or non-threatening social encounters may be misinterpreted as intrusive or hostile, reinforcing paranoid ideation. Considering the existing literature and our results, the orbitofrontal cortex seems to represent a critical neural marker to maintain personal space, and the hypoactivation observed in patients with paranoia might sustain a deficiency in regulatory mechanism.

Downregulation of negative emotion regulation (similar to those triggered by the experience of threat approaching) has been associated with increased BOLD signal in dlPFC, but also in posterior cingulate^43^. Thus, altered activation in these regions might further prevent adequate regulation of negative emotions in our patients with paranoia.

Furthermore, a study stimulating the ventro-lateral PFC showed reduction of paranoia symptoms in healthy individuals^13^. These results, although modest, confirm evidence for the involvement of lateral PFC in paranoia and offer promises as a potential treatment target. A recent randomized clinical trial by Fan et al. (2025) demonstrated that transcranial direct current stimulation (tDCS) applied to the VLPFC significantly reduced paranoia and improved social functioning in individuals with schizophrenia. This study employed a double-blind, within-subjects, crossover design with 50 participants, revealing that active tDCS sessions led to greater reductions in state paranoia and associated social cognitive biases compared to sham sessions. These findings suggest that VLPFC-targeted tDCS may serve as a promising intervention for mitigating paranoia and enhancing social outcomes in clinical populations^44^.

### Increased hippocampal activity during personal space violation

In a review from 2010 abnormal hippocampus activity during emotional stimuli processing was associated with greater positive symptoms, specifically paranoia^1^. The hippocampus has been linked to regulation of affective states, such as generating behaviors in threatening or potentially threatening contexts^45^, and the parahippocampal gyrus—closely connected to the hippocampus, has a role in context appraisal^46^. Impairments in the hippocampus might lead to abnormal emotion recognition, misinterpretation of neutral or ambiguous situations as threatening, and a reduced ability to regulate affective states, which in accordance with our finding, could lead to persecutory delusions^47^. Recent evidence suggested a direct link between altered hippocampus connectivity and paranoia in schizophrenia patients^11^ and hippocampus perfusion was associated with delusions in healthy individuals^48^. Further, positive schizotypy symptoms in a healthy population were associated with greater right hippocampal activation during an emotional Stroop task^49^. Additionally, levels of delusional thinking and associated levels of distress were correlated with hippocampal perfusion in another healthy cohort^48^, suggesting that similar to schizophrenia, non-help seeking individuals showed elevated activity in the hippocampus, reinforcing the hypothesis of a continuum of paranoia.

The framework of dopamine dysregulation leading to disrupted salience processing and paranoid symptoms^50,51^ further highlight the key role of the hippocampus, notably through the loss of parvalbumin interneurons (PVI) in the anterior hippocampus^52^. We could thus confirm previous evidence suggesting an association between hippocampal dysfunction and the severity of paranoia symptoms.

### Decreased activity in cerebellum and temporo-parietal regions

We further observed decreased activity both in the categorical approach and as a function of severity of paranoia in the posterior cerebellum, in the fusiform face area and temporo-parietal regions. Although little is known about cerebellar contributions to delusions, one study showed cerebellar deficits, predominantly linked to sensorimotor processing associated with delusional disorders presenting with somatic content^53^. Thus, we could hypothesize that paranoia severity modulates how motor actions are perceived during the task^54^. Additionally, the role of the cerebellum in social cognition has been increasingly recognized, and impaired sequencing and prediction mechanisms are thought to affect social abilities in schizophrenia^55^. The latter suggests that delusions symptoms may arise from suboptimal operation of predictive mechanisms involved in suppressing re-afferent feedback^55^. Further, face processing is crucial for social interaction, and schizophrenia patients showed specific impairments in the fusiform face area^56^, suggesting that patients with higher levels of paranoia may use less resources to understand the emotions they perceived during the task. Recent studies have further elucidated the role of the temporoparietal junction (TPJ) in personal space regulation and its implications for paranoia. Research indicates that abnormalities in personal space perception in psychosis involve disrupted function and connectivity of the TPJ and associated networks. This disruption may contribute to the altered social cognition observed in paranoia, where individuals misinterpret others’ intentions during close interpersonal interactions^57^. Additionally, the right TPJ (rTPJ) is crucial for distinguishing self from others, a process essential for appropriate personal space regulation. Impairments in this self-other distinction can lead to difficulties in social interactions, potentially resulting in paranoid ideation. For instance, studies suggest that deficits in self-other control mechanisms, mediated by the rTPJ, are associated with increased personal distress and paranoia^58^. Furthermore, evidence suggests that the right TPJ plays a direct role in perspective-taking, a key component of social cognition. Dysfunction in this region may contribute to paranoia by impairing the ability to infer others’ intentions accurately^59^. These findings suggest that hypoactivation or dysfunction of the TPJ may underlie the deficits in social cognition and personal space regulation observed in individuals with paranoia. Understanding the TPJ’s role offers potential avenues for targeted interventions aimed at improving social functioning in this population^60^.

Paranoia severity was associated with decreased activity in fronto-parietal regions and putamen when comparing retreat to static condition, while fronto-parietal decreased activity was observed in the categorical approach during approach vs static. These results echoed with the study from Holt and colleagues^36^ showing that both controls and patients with schizophrenia showed fronto-parietal responses but specifically to approaching vs withdrawing faces. Altogether, parietal-frontal circuit alterations supporting the sensory-guided initiation of behavior (including those occurring in personal space) may play a role in social dysfunction observed in paranoia. Corroborating these findings, Wisner and colleagues observed that a left fronto-parietal network was sensitive to rational mistrust, suggesting that these regions provide broad executive control of functioning^61^. Further, a recent study detected differences in texture parameters for the putamen indicating that this nucleus might be at stake in delusional disorders^62^. Although our results identified regions dysfunction linked to social behaviors and delusional disorders, it was in the context of approaching or withdrawal of social stimuli, thus, we could hypothesize that individuals with higher paranoia severity might also experience difficulties in executive control of behaviors in relation to retreating threatening stimuli.

### Absence of involvement of the amygdala during personal space violation

Although we expected increased activity of the amygdala during PS violation, our results did not show significant results regarding this region. Paranoia has been linked to increased resting cerebral blood flow activity of the amygdala, suggesting that baseline amygdala hyperactivity may be an inherent neurobiological mechanism of paranoid ideation^9^^,^^63^. Further, a recent study observed that participants without persecutory beliefs showed greater response in amygdala during a fear conditioning procedure, while participants with persecutory beliefs failed to exhibit this response^64^. Thus, we could suggest that patients with higher levels of paranoia, who already have a heightened resting state activity of the amygdala at baseline, might not be able to recruit this region in the context of the approaching fMRI task^65^.

#### Extending our results to other aspects of paranoia

Recent research on peripersonal space and self-other boundaries in schizophrenia^66^ suggests that difficulties in distinguishing the self from others may underlie various paranoia-related experiences, not just persecutory delusions. Their findings indicate that alterations in personal space regulation could be relevant to broader paranoid phenomena, including heightened sensitivity to social cues and increased mistrust. Similarly, our results suggest that the neural patterns we observed may reflect disruptions in social threat perception that extend beyond persecutory delusions, potentially contributing to ideas of reference and increased social vigilance.

By considering both dimensional and categorical frameworks, our study provides a more comprehensive and nuanced perspective on how neural dysfunctions in personal space processing might be relevant to different forms of paranoia. The categorical approach allowed us to identify group-level differences, while the dimensional approach helped capture th**e** continuum of paranoia severity, ensuring that our findings are not restricted to the most severe expressions of paranoia. This strengthens the interpretation that abnormalities in personal space processing may be a transdiagnostic feature of paranoia rather than being exclusive to persecutory delusions.

#### Absence of effect of emotional valence

The absence of significant group differences in the Angry > Neutral contrast suggests that paranoia-related neural alterations are not strongly driven by general emotional valence effects but rather by the interaction between emotional valence and approach behavior. The hypoactivity in the cerebellum and middle frontal gyrus in patients could reflect disruptions in movement observation, but these findings do not indicate a clear paranoia-specific effect. The regression analyses further reinforce this interpretation, as none of the emotional valence contrasts yielded significant associations with paranoia severity, suggesting that paranoia-related neural alterations are more pronounced in the context of social approach rather than isolated differences in processing emotional facial expressions.

These results collectively suggest that emotional valence alone does not significantly modulate paranoia-related neural activity, reinforcing our decision to focus on approach behaviour as the primary contrast of interest.

The absence of an effect of emotional valence on approach behavior contrasts with prior findings suggesting that emotional expressions—particularly anger—modulate approach-avoidance tendencies^67^. Studies on trustworthiness ratings indicate that angry faces are often perceived as less trustworthy and more threatening, which can bias approach tendencies^68^. Given this, one might have expected angry expressions to enhance avoidance or influence approach behavior, as previously observed when trustworthiness ratings predict differential responses to faces. Additionally, Haut & MacDonald^69^ found that while patients’ overall trustworthiness ratings of unfamiliar faces did not differ from controls, the correlation between trustworthiness and attractiveness judgments was significantly reduced. This suggests that social cues, such as facial attractiveness, may contribute to trustworthiness assessments, and disruptions in this process may be linked to specific psychopathologies.

In our study, several factors could explain the lack of an interaction effect between emotional valence and approach behavior. First, our task likely engages motor-driven responses, which may be less sensitive to social appraisal mechanisms like trustworthiness. Second, individual differences—such as vulnerability to persecutory delusions or other psychopathologies—might moderate the influence of emotional expressions on approach-avoidance behaviors but were not explicitly modeled in our analyses. Third, contextual factors, including task framing and movement execution demands, could have attenuated the expected effects of emotional valence.

In summary, the findings of the present study revealed that paranoia severity as measured with the GPTS-B was associated with increased activity in the right hippocampus, reinforcing the hypothesis of an alteration of the limbic system associated with paranoid delusions. Further, altered activity in a network encompassing dlPFC, cingulate and fronto-parietal areas might reflect PS regulation impairments.

### Limitations

Given that PS is a complex behavior depending upon several factors, we tried to account for the main variables previously reported in existing literature (age, sex), however, other factors might also influence the results (i.e., level of anxiety, social context), and should be accounted for in future research. Further studies might also include factors such as stress-related features (cortisol and reactivity measures) as they might also have an impact on preferred distance. Most of our patients were medicated, which, in principle, can alter neural activity. However, including OLZ equivalence dosage as covariate of interest in our supplementary analyses showed that the overall effect observed in our study persists even after accounting for medication differences (hypoactivation of the OFC in patients with high paranoia when compared to patients with low paranoia). Furthermore, the fMRI task has primarily investigated PS from a passive perspective (i.e., participants watched faces approaching them). PS is a social behavior that entails both letting others approach us as well as approaching others. In future studies, it might be of interest to combine neuroimaging with virtual reality to investigate both active and passive PS regulation^70^. While we ensured task engagement by asking all participants post-experiment whether they had noticed the approaching faces and maintained attention throughout, objective tracking of gaze fixation would have provided stronger evidence of attention allocation differences between groups. Additionally, subjective threat ratings during or immediately after the task would have enabled us to better quantify individual differences in threat perception. Prior research^71^ has demonstrated the importance of eye-tracking in understanding attentional biases in paranoia, showing that individuals with high paranoia symptoms tend to avoid direct eye contact and exhibit altered gaze patterns when processing social stimuli. Future studies incorporating these behavioral measures will help clarify the impact of variations in visual attention on brain imaging findings on subjective threat experience. Lastly, all participants in our study were white Caucasian Swiss men and women, ensuring homogeneity in cultural background, which is known to affect personal space preferences. Additionally, all stimuli consisted of white men and women, minimizing potential cross-ethnic effects in interpersonal distance perception. Future studies should aim to include participants from different ethnic and cultural backgrounds to explore how interpersonal space regulation varies across populations.

## Conclusion

We clarified which brain regions and functional connections impacted impaired PS regulation in schizophrenia patients experiencing current paranoid threat. Although our results did not speak directly to previous findings on varying population samples, we identified specific neural correlates (increased activity of the hippocampus, decreased activity of the OFC) of approaching social threat in schizophrenia patients with current persecutory ideation.

## Methods

### Participants

Participants included in this study were part of a larger project “Interpersonal Space Study in Schizophrenia”, *n* = 135 from which 93 individuals (Controls: *n* = 35; Patients: *n* = 58) took part in the fMRI approaching faces task. Patients with schizophrenia spectrum disorders diagnosed according to DSM-5 criteria (schizophrenia, schizoaffective disorder, schizophreniform disorder) were recruited from the in- and out-patient departments of the University Hospital of Psychiatry and Psychotherapy in Bern, and healthy controls from advertisement and words of mouth. Healthy controls were matched for age and sex and all participants signed written informed consent. The study protocol was approved by the local ethics committee (KEK 2016-00166) and followed the declaration of Helsinki. Exclusion criteria for all participants included substance use disorders (except nicotine), head trauma with concurrent loss of consciousness, history of neurologic disease and/or electroconvulsive treatment, and any MRI contraindication. Further exclusion criteria for controls included a history of psychiatric disorder, and first-degree relatives with schizophrenia or schizoaffective disorder. See Table 1 for demographic and clinical information.

### Clinical assessment

Current psychopathology was assessed with the Positive And Negative Syndrome Scale (PANSS^72^,), and symptoms history using the Comprehensive Assessment of Symptoms and History (CASH,^73^). All but 7 patients were taking antipsychotic medication; we calculated mean daily Olanzapine equivalents (OLZ,^74^).

Paranoia within both the control and patient groups was assessed using the Green et al. Paranoid Thoughts Scale (GPTS), consisting of two subscales assessing ideas of reference (GPTS-A) and ideas of persecution (GPTS-B)^75,76^. Three patients were excluded from the final sample because of missing GPTS data, and 11 participants (controls, *n* = 5 patients, *n* = 6) were excluded because of excessive head motion during the scanning session (>3 mm of translation and/or >3 degrees of rotation) leading to a total of *n* = 79, including 30 controls (mean age (sd) = 34.8(12.1)), *n* = 49 patients (mean age(sd) = 37.6(13.7)).

Regarding the categorical approach, the GPTS-B cut off (score >35) was used to separate patients in high (SZ-high-paranoia) and low (SZ-low-paranoia) paranoia groups. We used the GPTS-B scale because the original GPTS-A (reference scale) contains problematic items that are not fully separable from the persecutory ideation scale and is thus not recommended as a stand-alone scale^75^. We selected this cutoff (>35) based on prior literature. Freeman et al., 2021 have demonstrated that this threshold effectively distinguishes between high and low paranoia^75^ individuals. They recommended cut-off for identifying moderately severe persecutory ideation is a score of 35 or above, representing 0.80 standard deviation above the level of paranoia in the population. This analysis compared 30 controls, 18 SZ-high-paranoia, and 31 SZ-low-paranoia. See Table 1.

For the continuous approach, we used GPTS-B continuous scores to represent paranoia severity among controls and patients (30 controls, and 49 patients with various degree of paranoia). See Table 1.

### fMRI paradigm

The event-related previously validated experiment^33^ consisted of two 10-minutes runs during which participants watched passively videos of faces in three different types of motion: approach, retreat, and static. Participant had normal or corrected-to-normal vision. The approaching condition was determined as a video zooming in to a factor of 2.75 (3 s), up to a point that only the region involving the mouth, and eyes could be seen. This gave the impression that the approaching person almost touched the participant. Each participant was shown the same faces and same emotions in randomized order. Target images were thus divided into six categories using a 3 (motions) x 2 (emotions) factorial design: i) Approach-Angry (AA), ii) Approach-Neutral (AN), iii) Retreat-Angry (RA), vi) Retreat-Neutral (RN), v) Static-Angry (SA), vi) Static-Neutral (SN). For more details, see supplementary Figure 1.

### fMRI data acquisition, preprocessing and first level analyses

Structural and functional images were acquired at the Translational Imaging Center Bern of sitem-insel on a 3 T Magnetom Prisma scanner (Siemens Healthcare, Erlangen, Germany). The acquisition protocol for the structural sequence was a T1-weighted MP2RAGE images with 176 slices, TR = 5000 ms, TE = 2.98 ms, flip angle = 4/5^o^, field of view = 256 mm, slice thickness = 1 mm. The fMRI acquisition consisted of 2 sequences of 10 min each and resulting in 1200 bold-oxygenation-level-dependent (BOLD) mbep2d images (TR = 1000 ms, TE = 37 ms, axial slices = 72, slice thickness = 2.5 mm, flip angle = 30^o^, field of view = 230 mm).

fMRI data were processed and analyzed using Statistical Parametric Mapping 12 (SPM12, Welcome Department of Parametric Mapping 12, Welcome Department of Neuroscience, London, UK) and Matlab 2023.a, and followed classic preprocessing steps, see *supplementary material*. Brain responses were analyzed at the first level per participant following the Generalized Linear model (GLM) approach; the six experimental conditions were entered in the model (AA, AN, RA, RN, SA, SN).

### fMRI second level analysis: categorical approach (high paranoia vs low paranoia vs controls)

In the second-level analyses, individual contrast images were included as full factorial design to determine significant brain activation in patients with low paranoia (SZ-low-paranoia) vs patients with high paranoia (SZ-high-paranoia) vs healthy controls.

Furthermore, for clarity and due to the a priori hypothesis that personal space intrusion was best modeled with the approaching condition we here focused on the contrast approach>static. All other contrasts are presented in the supplementary material: controls vs SZ-low-paranoia, as well as all other conditions approach vs retreat, retreat vs static, and emotional valence).

### fMRI second level analysis: continuous approach (paranoia severity)

In the second-level analyses, a whole brain multiple regression model was run in SPM to identify brain regions which demonstrated correlated activation with paranoia symptoms severity (GPTS-B continuous) contrasting between conditions Approach > Static condition (primary analysis). Baseline associations were modeled by the Static Neutral condition.

### Secondary fMRI analyses—emotional valence

To investigate the effects of emotional valence on brain activation, secondary analyses were conducted on task-related contrasts involving angry and neutral facial expressions. Group-level comparisons were performed at the whole-brain level for the *Angry > Neutral* contrast comparing both patient subgroups (SZ-high-paranoia vs. SZ-low-paranoia) and the combined patient group vs. healthy controls.

Additionally, voxel-wise multiple regression analyses were carried out using paranoia severity (GPTS-B scores) as a continuous predictor across participants. Contrasts examined in these models included *Retreat > Static*, *Angry > Neutral*, *Approach Angry* *>* *Approach Neutral*, and *Approach Angry > Static Angry*. These analyses aimed to assess dimensional relationships between task-related brain activation and paranoia severity across diagnostic groups.

As an exploratory step, we conducted an additional regression analysis on the *Approach Angry* *>* *Static Neutral* contrast. This analysis was not corrected for multiple comparisons and was intended to probe potential patterns of activation that may have been missed by the pre-registered contrasts. Findings from this analysis are reported descriptively in the supplementary material.

For all statistical analyses, we chose a primary cluster-forming threshold of *p* = 0.001 and a cluster-wise correction (qFDR) for multiple comparisons at *p* < 0.05 was applied.

### Covariates

In all analyses, we included age, sex, and Total Intracranial Volume (TIV) as covariates of no interest. When comparing patient groups (SZ-high-paranoia vs SZ-low-paranoia), we added duration of illness as a covariate of no interest. See *supplementary material* for justification.

## Supplementary information

Supplementary Material

## Data Availability

Data from individuals included in the present study can be made available upon request. No codes were used in the present study.
