# Supplementary material for: I fear you’re getting too close: neural correlates of personal space violation in paranoia
Source: Schizophrenia (Heidelb). 2025 May 21;11(1):77. doi: 10.1038/s41537-025-00625-x (PMC12092760; doi:10.1038/s41537-025-00625-x)
Supplement: Supplementary file 1 — Supplementary Material [file 41537_2025_625_MOESM1_ESM.pdf]

Supplementary material

1. Supplementary Methods 3
1.1. The fMRI approaching faces task 3
1.2. fMRI analyses 4
1.3. Justification for covariates inclusion 5
1.4. Post-Hoc Power analysis 5
1.5. Demographics comparisons between the MRI subsample and the recruited sample 6
2. Supplementary Results 7
2.1. Repartition of GPTS-B between groups 7
2.2. fMRI supplementary results of categorical approach (covariates Age, Sex, TIV) 9
2.2.a. Group results - baseline (approach neutral) 9
2.2.b. Group results - Approach > Static, supplementary group comparisons 11
2.2.c. Group results - Approach > Retreat 11
2.2.d. Group results - Retreat < Static 12
2.2.e. Group results - Angry > Neutral 13
2.2.f. Group results - Approach Neutral > Static Neutral 14
2.2.g. Group results - Approach Angry > Static Angry 16
2.2.h. Group results - Static Angry > Static Neutral 18
2.3. fMRI supplementary results of continuous approach, multiple regression between paranoia severity and task conditions (covariates Age, Sex, TIV) 20
2.3.a. Regression results Approach > Retreat 20
2.3.b. Regression results Approach Neutral > Static Neutral 20
2.3.c. Regression results Static Angry > Static Neutral 21
2.3.d. Regression results Other contrasts 22

2.4. fMRI supplementary results of categorical approach and continuous approach looking at within patients only ... 22

2.4.a. Group differences between patients (SZ-high-paranoia vs SZ-high-paranoia) with Medication as covariate for the contrast of intesrest Approach>static ... 22

2.4.b. Group differences between patients (SZ-high-paranoia vs SZ-high-paranoia) with PANSS total as covariate for the contrast of intesrest Approach>static ... 23

2.5. fMRI supplementary results of categorical approach and continuous approach for the contrast Aproach>Static with TIV ony as a covariate ... 24

2.5.a. Categorical approach (Approach > Static) ... 24

2.5.b. Continuous approach (Approach > Static) ... 26

Supplementary References ... 27

1. Supplementary Methods

1.1. The fMRI approaching faces task

Supplementary Figure 1. fMRI task paradigm

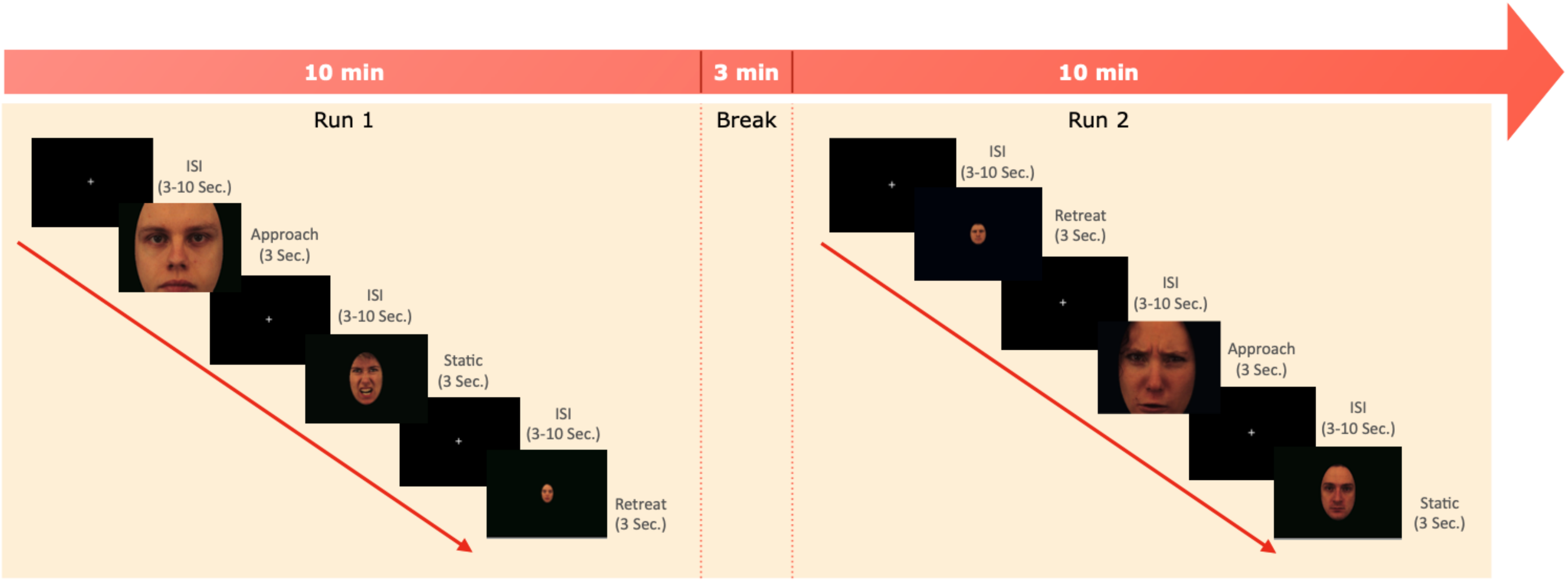

note. The experiment consisted in two 10-minutes runs during which participants watched passively videos of faces in three types of motion: approach, static, retreat. Pictures represented both males and females showing either angry or neutral emotional expression.

Each video was shown for 3 seconds. The approach condition consisted in a video zoomed in, retreat as zoomed out, and the static condition was only the projection of a picture.

The 3-minutes break between the two runs was used for attentional purposes. Pictures were taken from the Karolinska Directed Emotional Faces (Lundqvist et al., 1998) and represented both Males (50%) or Females (50%) showing either Angry or Neutral facial expressions. In total, participants were presented with 120 stimuli, and each video was shown for 3 seconds. The retreat condition was defined as zooming out until a factor of 0.2. The static condition was only the projection of a picture. A black background with a white cross was presented before each stimulus. During each 10 minutes run, 60 videos were presented for 3 seconds, with inter-trial intervals varying between 3000 and 10000 ms. Jitter times were pseudorandomized, and the two runs were counterbalanced between participants. The rate of facial stimuli was equal for all conditions, and participants were presented the same faces and emotional valence.

1.2. fMRI analyses

Functional MRI preprocessing was conducted using SPM12 and the CAT12 toolbox within MATLAB, following standard pipelines. Background-suppressed images were generated from MP2RAGE sequences using SPM's imcalc. Anatomical images were segmented using CAT12, and functional images were normalized to MNI152 space with 2mm isotropic voxels. Distortion correction was performed using fieldmap-based methods, followed by realignment and motion correction using a rigid-body transformation. Functional images were coregistered to anatomical images and smoothed with a 6mm full-width at half maximum Gaussian kernel.

All preprocessing was conducted in MATLAB (R2022a) following best practices for fMRI analyses. The six movement parameters were included as additional regressors of no interest in the design matrix. Using a threshold of 3 mm translation and 3º of rotation regarding the excessive head motion (Li et al., 2020; Stripeikyte et al., 2021), we excluded 11 participants. Furthermore, changes in the BOLD signal were investigated using the estimated GLM parameters for each contrast of interest.

1.3. Justification for covariates inclusion

We used TIV as a covariate of no interest to remove variability in head sizes. It corresponded to the sum of volumes extracted from the segmentation (white matter, grey matter, and cerebrospinal fluid). In addition we added sex and age as covariates of no interest as these variables were found to influence topological organization of the brain (Foo et al., 2021). Studies have shown that extended periods of relapse may negatively affect brain integrity in schizophrenia, underscoring the importance of considering illness duration in neuroimaging analyses (Andreasen et al., 2013). Thus, by controlling for duration of illness, we aim to isolate the effects of paranoia on neural activation from those related to the chronicity of the disorder. This approach enhances the validity of our findings by ensuring that observed neural differences are more likely attributable to paranoia levels rather than the progression of schizophrenia itself.

1.4. Post-Hoc Power analysis

We conducted a post-hoc power analysis using G*Power to evaluate the sensitivity of our study design for detecting differences in GPTS-B scores among controls, patients with paranoia, and patients without paranoia. The analysis, based on group sample sizes of

18, 30, and 31, yielded a post-hoc power of 0.371 for detecting a medium effect size (δ=0.5) with an alpha level of 0.05. While this is often referred to as a "post-hoc" power analysis, it essentially reflects the a priori power that would be expected for detecting such an effect with these sample sizes, rather than the power of the observed result.
We acknowledge that post-hoc power analyses have limited interpretability and do not alter the statistical inference drawn from the data. Moreover, given that our neuroimaging analyses used a conservative voxel-wise alpha threshold, actual power was likely even lower—further underscoring the challenges in detecting moderate effects with this sample.

Nonetheless, this preliminary effect size may serve as a useful starting point for powering future studies. To mitigate some of these limitations, we employed both categorical and continuous approaches in our analysis. This methodological choice allowed us to capture variability in paranoia severity beyond strict group categorizations, increasing the sensitivity of our findings. The continuous approach ensures that even if the three-group comparison lacks sufficient power for moderate effect sizes, we still retain the ability to detect meaningful associations across the full paranoia spectrum. Future studies with larger sample sizes will be necessary to confirm the current findings.

1.5. Demographics comparisons between the MRI subsample and the recruited sample

The MRI subsample reported here (n=93) did not significantly differ in demographics or paranoia severity from the larger sample (n=135-93=42).

MANOVAs

	Dependent Variable	Sum of Squares	df	Mean Square	F	p
**Group-MRI**	**OLZ_24hrs**	9.08	1	9.08	0.0745	0.786
	**Age_Years**	3.88	1	3.88	0.0248	0.875
	**Education_Years**	7.04	1	7.04	0.8897	0.349
	**GPTS_A_SUM**	334.53	1	334.53	1.2946	0.259
	**GPTS_B_SUM**	1092.46	1	1092.46	3.1419	0.080
	**PANSS_Pos**	8.81	1	8.81	0.2743	0.602
	**PANSS_Neg**	23.94	1	23.94	0.6708	0.415
	**PANSS_Total**	2.75	1	2.75	0.0115	0.915
	**duration_of_illness_max**	22.08	1	22.08	0.2910	0.591

2. Supplementary Results

2.1. Repartition of GPTS-B between groups

Supplementary figure 2

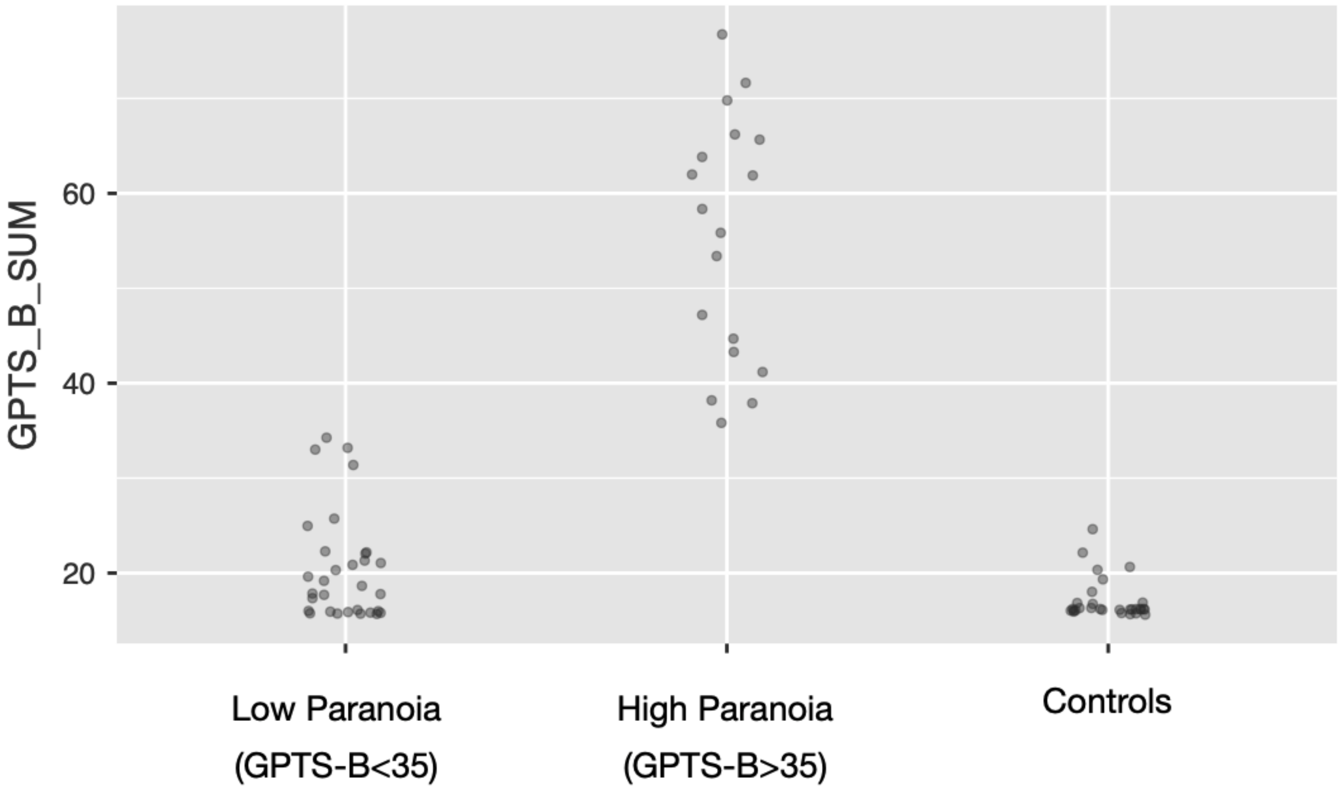
GPTS_B_SUM
60
40
20
Low Paranoia
(GPTS-B<35)
High Paranoia
(GPTS-B>35)
Controls

note. The grouping low and high paranoia was computed across schizophrenia patients for information. To do so we used the threshold of 35 for GPTS-B, accepted and validated in previous literature (Green et al., 2008). Here we show the repartition of GPTS-B scores, and we can observe the continuous repartition of such scores. This reinforces the use of GPTS-B as a continuous variable of paranoia severity along the manuscript.

2.2. fMRI supplementary results of categorical approach (covariates Age, Sex, TIV)

2.2.a. Group results - baseline (approach neutral)

	**p(FWE-corr)**	**p(FDR-corr)**	**Number of voxels**	**p(unc)**	**p(FWE-corr)**	**p(FDR-corr)**	**T**	**Z**	**p(unc)**	**x,y,z {mm}**	**Region name**
**Controls > Patients**	nothing										
**Controls < Patients**											
	0.065	0.035	52	0.001	0.598	0.807	4.89	4.53	0.001	-20 -84 4	L- inf occ gyrus
					1	0.959	3.26	3.14	0.001	-28 -76 0	L- occ fusiform gyrus
	0.027	0.028	62	0.001	0.999	0.959	4.2	3.97	0.001	30 -68 -32	R-cerebellum
					1	0.959	4.05	3.84	0.001	36 -62 -36	R-cerebellum
**Controls > SZ-high-paranoia**	nothing										
**Controls< SZ-high-paranoia**											

	0.001	0.001	135	0.001	0.218	0.452	5.25	4.82	0.001	-20 -84 4	L inf occ gyrus
					1	0.858	3.97	3.77	0.001	-28 -76 0	L- occ fusiform gyrus
					1	0.858	3.93	3.73	0.001	-2 -64 0	L- lingual gyrus
	0.012	0.005	72	0.001	0.757	0.452	4.75	4.42	0.001	28 28 38	R- middle frontal gyrus
					1	0.858	3.93	3.73	0.001	18 22 42	R- supp frontal gyrus
	0.007	0.005	79	0.001	0.794	0.452	4.72	4.39	0.001	-16 0 36	L middle cingulate gyrus
					1	0.711	4.16	3.93	0.001	-20 -4 44	L supp frontal gyrus
**Controls > SZ-low-paranoia**											
	0.025	0.008	63	0.001	0.953	0.984	4.5	4.22	0.001	-8 -88 -6	L- limgual gyrus
					1	0.984	3.29	3.16	0.001	-14 -96 -2	L occipital pole
	0.038	0.008	58	0.001	1	0.984	3.89	3.7	0.001	44 20 26	R middle frontal gyrus
					1	0.984	3.33	3.2	0.001	52 18 26	R oppercular part of inf frontal gyrus
**Controls < SZ-low-paranoia**	nothing										
**SZ-high-paranoia > SZ-low-paranoia**											
	0.001	0.002	99	0.001	0.119	0.287	5.4	4.94	0.001	-30 10 40	L- middle frontal g
					0.932	0.81	4.54	4.25	0.001	-32 16 50	L- middle frontal g
	0.003	0.002	90	0.001	0.438	0.338	5.03	4.65	0.001	-10 0 34	L- middle cingulate G
					1	0.921	4.05	3.83	0.001	-18 -4 42	L- middle cingulate G
	0.023	0.011	64	0.001	0.903	0.81	4.59	4.29	0.001	-64 -18 2	L- sup temporal g
					1	0.921	3.74	3.57	0.001	-64 -20 10	L- planum temporal
**SZ-high-paranoia < SZ-low-paranoia**	nothing										

2.2.b. Group results - Approach > Static, supplementary group comparisons

	p(FWE-corr)	p(FDR-corr)	Number of voxels	p(unc)	p(FWE-corr)	p(FDR-corr)	T	Z	p(unc)	x,y,z {mm}	Region name
**Controls > Patients**											
	0.001	0.001	1737	0.001	0.021	0.08	5.83	5.27	0.001	4 -82 24	R cuneus
					0.532	0.336	4.95	4.58	0.001	-8 -74 -6	L lingual g
					0.703	0.448	4.8	4.47	0.001	-2 -80 2	L lingual g
	0.001	0.001	221	0.001	0.065	0.109	5.55	5.06	0.001	12 -42 48	R-precuneus
					1	0.788	3.62	3.46	0.001	-6 -42 56	L-precuneus
	0.001	0.001	140	0.001	0.446	0.327	5.02	4.65	0.001	-50 -66 4	L MTG
					1	0.788	3.59	3.44	0.001	-54 -62 -2	L MTG
	0.001	0.001	100	0.001	1	0.707	4.16	3.93	0.001	42 -40 -22	R fusiform
					1	0.902	3.38	3.25	0.001	52 -46 -20	R ITG
**Controls < Patients**	nothing										
**Controls > SZ-low-paranoia**											
	0.001	0.001	156	0.001	1	0.941	4.06	3.85	0.001	2 -82 0	L lingual G
					1	0.941	3.93	3.73	0.001	-10 -74 -6	L lingual G
					1	0.956	3.58	3.43	0.001	-6 -82 -2	L lingual G
**Controls < SZ-low-paranoia**	nothing										

2.2.c. Group results - Approach > Retreat

**Controls > Patients**	**p(FWE-corr)**	**p(FDR-corr)**	**Number of voxels**	**p(unc)**	**p(FWE-corr)**	**p(FDR-corr)**	**T**	**Z**	**p(unc)**	**x,y,z {mm}**	**Region name**
	0.001	0.001	1138	0.001	0.438	0.374	5.01	4.64	0.001	2 -86 20	L cuneus
					0.967	0.602	4.45	4.18	0.001	0 -82 0	L lingual G
					1	0.702	4.18	3.95	0.001	-10 -78 22	L cuneus
**Controls < Patients**	nothing										
**Controls > SZ-high-paranoia**											
	0.001	0.001	1523	0.001	0.25	0.401	5.22	4.8	0.001	4 -82 24	R cuneus
					0.741	0.481	4.75	4.42	0.001	-2 -84 12	L cuneus
					0.754	0.481	4.74	4.41	0.001	-8 -78 22	L cuneus
**Controls< SZ-high-paranoia**	nothing										
**Controls > SZ-low-paranoia**											
	0.001	0.001	114	0.001	1	0.926	3.97	3.77	0.001	0 -84 0	L lingual G
					1	0.926	3.35	3.22	0.001	-6 -80 -6	L lingual G
					1	0.953	3.3	3.17	0.001	8 -88 0	R calcarine
**Controls < SZ-low-paranoia**	nothing										
**SZ-high-paranoia >SZ-low-paranoia**	nothing										
**SZ-high-paranoia < SZ-low-paranoia**	nothing										

2.2.d. Group results - Retreat < Static

**Controls > Patients**	**p(FWE-corr)**	**p(FDR-corr)**	**Number of voxels**	**p(unc)**	**p(FWE-corr)**	**p(FDR-corr)**	**T**	**Z**	**p(unc)**	**x,y,z {mm}**	**Region name**

	0.009	0.002	78	0	0.914	0.698	4.56	4.26	0	-48 -66 4	L MTG
**Controls < Patients**	nothing										
**Controls > SZ-high-paranoia**	nothing										
**Controls< SZ-high-paranoia**	nothing										
**Controls > SZ-low-paranoia**											
	0	0	150	0	0.264	0.087	5.2	4.79	0	-48 -66 4	L MTG
**Controls < SZ-low-paranoia**	nothing										
**SZ-high-paranoia > SZ-low-paranoia**	nothing										
**SZ-high-paranoia < SZ-low-paranoia**	nothing										

2.2.e. Group results - Angry > Neutral

**Controls > Patients**	**p(FWE-corr)**	**p(FDR-corr)**	**Number of voxels**	**p(unc)**	**p(FWE-corr)**	**p(FDR-corr)**	**T**	**Z**	**p(unc)**	**x,y,z {mm}**	**Region name**
	0.258	0.084	37	0.003	0.766	0.796	4.74	4.41	0	10 -68 -30	Cerebellum
	0.08	0.047	50	0.001	0.964	0.796	4.47	4.19	0	22 38 18	R MFG
**Controls < Patients**	nothing										
**Controls > SZ-high-paranoia**	nothing										
**Controls< SZ-high-paranoia**	nothing										
**Controls > SZ-low-paranoia**	nothing										

**Controls < SZ-low-paranoia**	nothing										
**SZ-high-paranoia > SZ-low-paranoia**	nothing										
**SZ-high-paranoia < SZ-low-paranoia**	nothing										

2.2.f. Group results - Approach Neutral > Static Neutral

	p(FWE-corr)	p(FDR-corr)	Number of voxels	p(unc)	p(FWE-corr)	p(FDR-corr)	T	equivZ	p(unc)	x,y,z {mm}	Region name
**Controls > Patients**	0.003	0.001	87	0.001	0.321	0.307	5.15	4.75	0.001	46 -44 32	R supramarginal
					0.996	0.537	4.32	4.06	0.001	52 -42 22	R STG
	0.001	0.001	851	0.001	0.423	0.307	5.05	4.67	0.001	4 -82 22	R cuneus
					0.781	0.422	4.74	4.41	0.001	-6 -78 -6	L lingual gyrus
					0.98	0.537	4.43	4.16	0.001	2 -80 0	R lingual gyrus
	0.001	0.001	209	0.001	0.447	0.307	5.03	4.65	0.001	10 -42 44	R cingulate gyrus
					1	0.701	4.1	3.88	0.001	10 -52 56	R precuneus
	0.006	0.002	79	0.001	0.974	0.537	4.45	4.18	0.001	-22 -84 8	L MOG
					1	0.838	3.76	3.58	0.001	-18 -94 14	L MOG
	0.018	0.004	65	0.001	0.997	0.543	4.29	4.04	0.001	0 -58 -40	L cerebellum
**Controls < Patients**	nothing										
**Controls > SZ-high-paranoia**											
	0.001	0.001	1439	0.001	0.007	0.04	6.11	5.48	0.001	4 -80 24	R cuneus
					0.955	0.576	4.51	4.22	0.001	-4 -76 -4	L linual gyrus
					0.957	0.576	4.5	4.22	0.001	0 -84 18	L Cuneus
	0.001	0.001	175	0.001	0.504	0.576	4.98	4.61	0.001	12 -42 48	R precuneus
					1	0.954	3.58	3.42	0.001	4 -50 54	R precuneus
	0.005	0.002	80	0.001	0.783	0.576	4.74	4.41	0.001	42 -42 -24	R cerebellum
					1	0.898	3.73	3.56	0.001	40 -34 -26	R cerebellum
					1	0.962	3.43	3.3	0.001	38 -40 -16	R fusiform gyrus
	0.001	0.001	103	0.001	0.978	0.576	4.44	4.16	0.001	52 -42 16	R IPL

					0.997	0.576	4.29	4.04	0.001	46 -44 32	R supramarginal gyrus
	0.007	0.008	76	0.001	0.996	0.576	4.32	4.06	0.001	-24 -86 8	L MOG
**Controls < SZ-high-paranoia**		nothing									
**Controls > SZ-low-paranoia**											
	0.001	0.07	111	0.001	1	0.948	3.76	3.58	0.001	2 -82 0	R lingual gyrus
					1	0.948	3.67	3.51	0.001	-8 -74 -6	L lingual gyrus
					1	0.948	3.43	3.29	0.001	-16 -76 -4	L lingual gyrus
**Controls < SZ-low-paranoia**		nothing									
**SZ-high-paranoia > SZ-low-paranoia**		nothing									
**SZ-high-paranoia < SZ-low-paranoia**											
	0.001	0.07	111	0.001	1	0.948	3.76	3.58	0.001	2 -82 0	R lingual gyrus
					1	0.948	3.67	3.51	0.001	-8 -74 -6	L lingual gyrus
					1	0.948	3.43	3.29	0.001	-16 -76 -4	L lingual gyrus

2.2.g. Group results - Approach Angry > Static Angry

**p(FWE-corr)**	**p(FDR-corr)**	**Number of voxels**	**p(unc)**	**p(FWE-corr)**	**p(FDR-corr)**	**T**	**equivZ**	**p(unc)**	**x,y,z {mm}**	**Region name**
**Controls > Patients**										
0.001	0.001	420	0.001	0.157	0.294	5.33	4.89	0.001	2 -86 22	R cuneus
				1	0.731	4.04	3.83	0.001	14 -90 8	R cuneus
				1	0.731	3.99	3.79	0.001	4 -86 32	R cuneus
0.001	0.058	135	0.001	0.874	0.731	4.64	4.33	0.001	-50 -66 4	L MTG
0.001	0.001	306	0.001	0.95	0.731	4.52	4.23	0.001	0 -80 2	R lingual G
				0.999	0.731	4.21	3.97	0.001	-8 -74 -6	L Lingual G
				1	0.731	3.89	3.7	0.001	4 -70 0	R lingual G

0.001	0.001	182	0.001	1	0.731	4.2	3.96	0.001	20 -70 20	R precuneus
				1	0.731	4.15	3.93	0.001	30 -80 24	R MOG
				1	0.731	3.88	3.69	0.001	32 -72 20	R precuneus
**Controls < Patients**										
nothing										
**Controls > SZ-high-paranoia**										
0.001	0.001	937	0.001	0.191	0.273	5.28	4.85	0.001	2 -86 24	R cuneus
				0.666	0.514	4.84	4.49	0.001	2 -78 42	R precuneus
				0.975	0.899	4.45	4.17	0.001	2 -66 18	R precuneus
0.001	0.001	187	0.001	0.553	0.39	4.93	4.57	0.001	56 -50 -22	R cerebellum
				0.601	0.39	4.89	4.54	0.001	46 -62 -12	R fusiform G
				1	0.899	4.12	3.9	0.001	52 -68 -12	R fusiform G
0.001	0.001	168	0.001	0.999	0.899	4.22	3.98	0.001	40 -38 56	R IPL
				1	0.899	4.03	3.82	0.001	28 -52 54	R SPL
				1	0.899	3.83	3.65	0.001	40 -46 58	R post central G
0.001	0.001	144	0.001	1	0.899	4.16	3.93	0.001	-6 -76 -6	L Lingual G
				1	0.899	3.88	3.69	0.001	0 -80 2	R lingual G
				1	0.899	3.7	3.54	0.001	6 -74 -2	R lingual G
0.008	0.036	75	0.001	1	0.899	3.73	3.56	0.001	32 -72 20	R precuneus
				1	0.899	3.7	3.54	0.001	30 -80 24	R MOG
				1	0.899	3.66	3.49	0.001	32 -84 16	R MOG
**Controls < SZ-high-paranoia**										
nothing										
**controls > SZ-low-paranoia**										
0.002	0.001	93	0.001	0.907	0.59	4.59	4.29	0.001	-46 -66 6	L MTG
0.009	0.008	73	0.001	0.996	0.59	4.31	4.06	0.001	-48 -80 14	L MOG
				1	0.59	4.12	3.9	0.001	-42 -86 14	L MOG

				1	0.901	3.6	3.45	0.001	-40 -78 12	L MOG
0.006	0.001	79	0.001	1	0.62	3.99	3.79	0.001	2 -86 22	R cuneus
				1	0.901	3.67	3.51	0.001	8 -88 14	R cuneus
				1	0.901	3.66	3.5	0.001	-2 -90 14	L cuneus
**Controls < SZ-low-paranoia**										
nothing										
**SZ-high-paranoia > SZ-low-paranoia**										
nothing										
**SZ-high-paranoia< SZ-low-paranoia**										
0.001	0.039	99	0.001	0.969	0.975	4.47	4.19	0.001	42 -42 58	R postcentral G
				1	0.983	3.71	3.54	0.001	44 -40 50	R IPL
0.001	0.001	219	0.001	0.994	0.975	4.34	4.08	0.001	48 -60 -16	R fusiform G
				0.997	0.975	4.3	4.05	0.001	50 -52 -20	R cerebellum
				1	0.975	4.02	3.81	0.001	58 -52 -12	R fusiform G

2.2.h. Group results - Static Angry > Static Neutral

p(FWE-corr)	p(FDR-corr)	Number of voxels	p(unc)	p(FWE-corr)	p(FDR-corr)	T	equivZ	p(unc)	x,y,z {mm}	Region name
**Controls > Patients**										
0.076	0.021	50	0.001	0.964	0.648	4.48	4.2	0	12 -72 -32	R cerebellum
0.012	0.005	71	0	0.967	0.648	4.47	4.19	0	-10 -74 -34	L cerebellum
				1	0.979	3.77	3.59	0	-16 -76 -40	L cerebellum
				1	0.988	3.58	3.43	0	-14 -80 -30	L cerebellum
0.002	0.002	92	0	0.988	0.648	4.38	4.12	0	24 -70 -46	R cerebelum
				1	0.886	4.18	3.94	0	30 -70 -40	R cerebellum
				1	0.979	3.73	3.56	0	30 -68 -32	R cerebellum

**Controls < Patients**										
nothing										
**Controls > SZ-high-paranoia**										
0.031	0.018	60	0	0.669	0.472	4.83	4.49	0	-12 2 32	L cingulate G
				0.941	0.472	4.53	4.24	0	-18 -4 38	L cingulate G
0.029	0.018	61	0	0.983	0.474	4.41	4.14	0	-10 -74 -34	L cerebellum
				1	0.681	4.13	3.9	0	-18 -80 -28	L cerebellum
				1	0.906	3.72	3.55	0	-16 -80 -36	L cerebellum
0.076	0.029	50	0.001	1	0.625	4.18	3.94	0	-28 -30 30	L cerebellum
				1	0.906	3.74	3.57	0	-32 -42 32	L supramarginal G
				1	0.906	3.57	3.42	0	-22 -22 32	L supramarginal G
**Controls < SZ-high-paranoia**										
nothing										
**Controls > SZ-low-paranoia**										
0.01	0.004	73	0	0.95	0.775	4.51	4.23	0	30 -66 -42	R cerebellum
**Controls < SZ-low-paranoia**										
noting										
**SZ-high-paranoia > SZ-low-paranoia**										
nothing										
**SZ-high-paranoia < SZ-low-paranoia**										
nothing										

2.3. fMRI supplementary results of continuous approach, multiple regression between paranoia severity and task conditions (covariates Age, Sex, TIV)

The regression results of baseline, Approach>static and Approach>retreat are presented in Table 3.

2.3.a. Regression results Approach > Retreat

When comparing approach to retreat conditions, paranoia severity was negatively associated with activation in the left thalamus, right occipital lobe (fusiform, inferior, superior and middle occipital gyri) and cerebellum. The positive correlation did not yield significant results. See Table 3.

2.3.b. Regression results Approach Neutral > Static Neutral

The positive regression did not yield significant results; however, paranoia severity was negatively associated with activation in the occipital and middle and superior frontal regions. See Table 2.3.b.

p(FWE-corr)	p(FDR-corr)	Number of voxels	p(unc)	p(FWE-corr)	p(FDR-corr)	T	equivZ	p(unc)	x,y,z {mm}	Region name
**Positive**										
nothing										hippocampus not significant
**Negative**										

0.001	0.001	137	0.001	0.583	0.614	4.9	4.55	0.001	4 -80 24	R cuneus
				1	0.897	3.84	3.66	0.001	-8 -76 22	L cuneus
				1	0.925	3.74	3.57	0.001	-2 -82 18	L cuneus
0.034	0.014	58	0.001	0.829	0.674	4.68	4.37	0.001	-32 8 40	L MFG
0.034	0.014	58	0.001	0.957	0.707	4.5	4.22	0.001	-52 28 10	L IFG
0.029	0.014	60	0.001	1	0.864	4.14	3.92	0.001	-16 -82 -36	L cerebellum
0.002	0.002	91	0.001	1	0.864	4.13	3.91	0.001	4 52 22	R MFG
				1	0.897	3.91	3.72	0.001	0 56 30	L SFG
				1	0.925	3.72	3.55	0.001	8 60 24	R SFG

2.3.c. Regression results Static Angry > Static Neutral

The positive regression did not yield significant results; however, paranoia severity was negatively associated with activation in the post central gyrus and cerebellum. See supplementary Table 2.3.c.

p(FWE-corr)	p(FDR-corr)	Number of voxels	p(unc)	p(FWE-corr)	p(FDR-corr)	T	equivZ	p(unc)	x,y,z {mm}	Region name
**Positive**										
nothing										
**Negative**										
0.075	0.054	50	0.001	0.825	0.648	4.68	4.37	0.001	-26 -32 30	L Postcentral G
				1	0.854	3.6	3.44	0.001	-30 -26 38	L Postcentral G
0.052	0.054	54	0.001	1	0.759	4.15	3.93	0.001	-18 -80 -28	L cerebellum
				1	0.854	3.99	3.79	0.001	-16 -82 -36	L cerebellum

2.3.d. Regression results Other contrasts

The other contrasts tested did not yield significant results, including Retreat > Static, Angry > Neutral, Approach Angry > Approach Neutral and Approach Angry > Static Angry. Exploratory result of the contrast Approach Angry > Static Neutral without multiple correction identified the same cluster (R-hippocampus) as for the main contrast of interest Approach>Static.

2.4. fMRI supplementary results of categorical approach and continuous approach looking at within patients only

2.4.a. Group differences between patients (SZ-high-paranoia vs SZ-high-paranoia) with Medication as covariate for the contrast of intesrest Approach>static

p(FWE-corr)	p(FDR-corr)	Number of voxels	p(unc)	p(FWE-corr)	p(FDR-corr)	T	equivZ	p(unc)	x,y,z {mm}	Region name
**Approach>static**										
**SZ-low-paranoia > SZ-high-paranoia**										
0.04	0.109	57	0	0.14	0.249	5.66	4.91	0	44 44 -14	R OFC BA11
				1	0.966	3.85	3.56	0	46 50 -8	R OFC BA10
**SZ-low-paranoia < SZ-high-paranoia**										
nothing										

2.4.b. Group differences between patients (SZ-high-paranoia vs SZ-high-paranoia) with PANSS total as covariate for the contrast of intesrest Approach>static

p(FWE-corr)	p(FDR-corr)	Number of voxels	p(unc)	p(FWE-corr)	p(FDR-corr)	T	equivZ	p(unc)	x,y,z {mm}	Region name
**Approach>static**										
**SZ-low-paranoia > SZ-high-paranoia**										
0	0	150	0	0.509	0.589	5.24	4.62	0	10 -66 -12	R cerebellum
				0.562	0.589	5.19	4.58	0	10 -62 -24	R cerebellum
				1	0.988	3.39	3.19	0.001	18 -62 -16	R cerebellum
0	0	267	0	0.707	0.589	5.05	4.48	0	44 -58 -20	R cerebellum
				0.994	0.606	4.52	4.09	0	38 -62 -24	R cerebellum
				0.995	0.606	4.51	4.08	0	40 -48 -28	R cerebellum
0	0	151	0	0.934	0.589	4.76	4.27	0	40 -40 4	R caudate (temporal lobe)
				0.951	0.589	4.72	4.24	0	52 -42 16	R STG
				0.999	0.606	4.38	3.98	0	56 -42 8	R STG
0.014	0.015	68	0	0.978	0.589	4.63	4.17	0	44 48 -14	R MFG, BA11
				0.998	0.606	4.44	4.03	0	38 40 -18	R MFG, BA11
				1	0.85	3.85	3.56	0	40 54 -10	R MFG, BA11
0	0	340	0	0.985	0.589	4.59	4.15	0	2 -66 18	R precuneus
				0.998	0.606	4.44	4.03	0	10 -76 16	R cuneus
				0.999	0.606	4.38	3.98	0	12 -60 10	R posterior cingulate
0.001	0.002	99	0	1	0.606	4.33	3.95	0	-2 -102 -2	R cuneus
				1	0.769	4.01	3.69	0	-6 -96 -12	L lingual G
				1	0.85	3.82	3.54	0	2 -94 -14	R Lingual G
**SZ-low-paranoia < SZ-high-paranoia**										
nothing										

2.5. fMRI supplementary results of categorical approach and continuous approach for the contrast Aproach>Static with TIV ony as a covariate

2.5.a. Categorical approach (Approach > Static)

Controls > SZ-high-paranoia										
p(FWE-corr)	p(FDR-corr)	Number of voxels	p(unc)	p(FWE-corr)	p(FDR-corr)	T	equivZ	p(unc)	x,y,z {mm}	Region name
0.001	0.001	1777	0.001	0.052	0.136	5.59	5.1	0.001	4 -82 24	R cuneus
				0.334	0.208	5.12	4.73	0.001	-8 -74 -6	
				0.459	0.226	5	4.64	0.001	-2 -80 2	
0.001	0.001	218	0.001	0.085	0.136	5.47	5.01	0.001	12 -42 48	R precuneus
				1	0.743	3.72	3.55	0.001	-6 -42 56	
0.001	0.001	158	0.001	0.365	0.208	5.09	4.71	0.001	-50 -66 4	L MTG
				1	0.754	3.64	3.48	0.001	-54 -62 -2	
0.018	0.005	66	0.001	0.599	0.289	4.88	4.54	0.001	22 -50 -14	R cerebellum
0.061	0.006	52	0.001	0.997	0.598	4.29	4.04	0.001	0 -54 -38	L cerebellum
				1	0.92	3.29	3.17	0.001	2 -60 -48	
0.004	0.001	83	0.001	0.998	0.598	4.26	4.02	0.001	20 -70 34	R precuneus
				0.999	0.598	4.25	4.01	0.001	16 -78 46	
0.003	0.001	89	0.001	0.999	0.598	4.23	4	0.001	42 -42 -24	R cerebellum
0.001	0	105	0.001	1	0.598	4.09	3.88	0.001	30 -82 20	R MOG
				1	0.754	3.67	3.51	0.001	24 -90 30	
				1	0.822	3.44	3.31	0.001	36 -76 22	

0.006	0.001	80	0.001	1	0.598	4.02	3.82	0.001	12 -54 64	R postcentral G
0.007	0.001	78	0.001	1	0.598	3.99	3.79	0.001	54 -42 22	R STG
				1	0.754	3.67	3.51	0.001	62 -42 18	
				1	0.822	3.46	3.32	0.001	64 -38 26	
0.015	0.005	68	0.001	1	0.714	3.8	3.62	0.001	-22 -88 8	L MOG
				1	0.809	3.53	3.38	0.001	-20 -92 16	
**SZ-high-paranoia< SZ-low-paranoia**										
0.027	0.042	61	0.001	0.138	0.482	5.35	4.92	0.001	44 44 -16	R orbito frontal G (BA11)
				1	0.9	4.11	3.89	0.001	46 50 -8	R orbito frontal G (BA10)
0.003	0.002	88	0.001	0.445	0.607	5.01	4.65	0.001	10 -66 -28	R cerebellum
				0.731	0.607	4.77	4.45	0.001	10 -64 -20	
				1	0.957	3.43	3.3	0.001	4 -68 -14	
0.001	0.001	192	0.001	0.925	0.9	4.55	4.27	0.001	4 -78 24	R cuneus
				1	0.9	4.05	3.84	0.001	10 -76 16	
				1	0.9	4.01	3.81	0.001	2 -64 16	
0.001	0.001	103	0.001	0.983	0.9	4.41	4.14	0.001	16 -60 4	R lingual G
				1	0.9	3.9	3.72	0.001	20 -68 6	
				1	0.957	3.46	3.33	0.001	18 -62 12	
0.019	0.042	65	0.001	0.983	0.9	4.41	4.14	0.001	52 -42 16	R STG
				1	0.922	3.7	3.54	0.001	56 -42 8	
0.018	0.005	66	0.001	1	0.9	4	3.8	0.001	36 -40 -28	R cerebellum
				1	0.922	3.67	3.51	0.001	46 -48 -20	
				1	0.922	3.62	3.47	0.001	34 -48 -24	
0.008	0.004	75	0.001	1	0.9	3.91	3.72	0.001	36 12 32	R Prcentral G
				1	0.9	3.82	3.64	0.001	44 14 34	

				1	0.922	3.74	3.57	0.001	50 10 38	

2.5.b. Continuous approach (Approach > Static)

**Positive correlation**										
**p(FWE-corr)**	**p(FDR-corr)**	**Number of voxels**	**p(unc)**	**p(FWE-corr)**	**p(FDR-corr)**	**T**	**equivZ**	**p(unc)**	**x,y,z {mm}**	**Region name**
0.229	0.032	38	0.003	0.987	0.447	4.37	4.12	0.001	28 -16 -10	R Hippocampus
**Negative correlation**										
0.077	0.154	50	0.001	0.948	0.992	4.5	4.23	0.001	4 -80 24	R cuneus
				1	0.993	3.24	3.12	0.001	0 -88 24	R cuneus

Supplementary References

[4] Nieto-Castanon, A. (2020). FMRI minimal preprocessing pipeline. In Handbook of functional connectivity Magnetic Resonance Imaging methods in CONN (pp. 3–16). Hilbert Press.

[5] Andersson, J. L., Hutton, C., Ashburner, J., Turner, R., & Friston, K. J. (2001). Modeling geometric deformations in EPI time series. Neuroimage, 13(5), 903-919.

[6] Friston, K. J., Ashburner, J., Frith, C. D., Poline, J. B., Heather, J. D., & Frackowiak, R. S. (1995). Spatial registration and normalization of images. Human brain mapping, 3(3), 165-189.

[7] Henson, R. N. A., Buechel, C., Josephs, O., & Friston, K. J. (1999). The slice-timing problem in event-related fMRI. NeuroImage, 9, 125.

[8] Sladky, R., Friston, K. J., Tröstl, J., Cunnington, R., Moser, E., & Windischberger, C. (2011). Slice-timing effects and their correction in functional MRI. Neuroimage, 58(2), 588-594.

[9] Whitfield-Gabrieli, S., Nieto-Castanon, A., & Ghosh, S. (2011). Artifact detection tools (ART). Cambridge, MA. Release Version, 7(19), 11.

[10] Power, J. D., Mitra, A., Laumann, T. O., Snyder, A. Z., Schlaggar, B. L., & Petersen, S. E. (2014). Methods to detect, characterize, and remove motion artifact in resting state fMRI. Neuroimage, 84, 320-341.

[11] Nieto-Castanon, A. (submitted). Preparing fMRI Data for Statistical Analysis. In M. Filippi (Ed.). fMRI techniques and protocols. Springer. doi:10.48550/arXiv.2210.13564

[12] Calhoun, V.D., Wager, T.D., Krishnan, A., Rosch, K.S., Seymour, K.E., Nebel, M.B., Mostofsky, S.H., Nyalakanai, P. and Kiehl, K. (2017). The impact of T1 versus EPI spatial normalization templates for fMRI data analyses (Vol. 38, No. 11, pp. 5331-5342).

[13] Ashburner, J., & Friston, K. J. (2005). Unified segmentation. Neuroimage, 26(3), 839-851.

[14] Ashburner, J. (2007). A fast diffeomorphic image registration algorithm. Neuroimage, 38(1), 95-113.

[15] Nieto-Castanon, A. (2020). FMRI denoising pipeline. In Handbook of functional connectivity Magnetic Resonance Imaging methods in CONN (pp. 17–25). Hilbert Press.

[16] Friston, K. J., Williams, S., Howard, R., Frackowiak, R. S., & Turner, R. (1996). Movement-related effects in fMRI time-series. Magnetic resonance in medicine, 35(3), 346-355.

[17] Hallquist, M. N., Hwang, K., & Luna, B. (2013). The nuisance of nuisance regression: spectral misspecification in a common approach to resting-state fMRI preprocessing reintroduces noise and obscures functional connectivity. Neuroimage, 82, 208-225.

[18] Behzadi, Y., Restom, K., Liau, J., & Liu, T. T. (2007). A component based noise correction method (CompCor) for BOLD and perfusion based fMRI. Neuroimage, 37(1), 90-101.

[19] Chai, X. J., Nieto-Castanon, A., Ongur, D., & Whitfield-Gabrieli, S. (2012). Anticorrelations in resting state networks without global signal regression. Neuroimage, 59(2), 1420-1428.

[20] Friston, K. J., Buechel, C., Fink, G. R., Morris, J., Rolls, E., & Dolan, R. J. (1997). Psychophysiological and modulatory interactions in neuroimaging. Neuroimage, 6(3), 218-229.

[21] McLaren, D. G., Ries, M. L., Xu, G., & Johnson, S. C. (2012). A generalized form of context-dependent psychophysiological interactions (gPPI): a comparison to standard approaches. Neuroimage, 61(4), 1277-1286.

[22] Nieto-Castanon, A. (2020). General Linear Model. In Handbook of functional connectivity Magnetic Resonance Imaging methods in CONN (pp. 63–82). Hilbert Press.

[23] Worsley, K. J., Marrett, S., Neelin, P., Vandal, A. C., Friston, K. J., & Evans, A. C. (1996). A unified statistical approach for determining significant signals in images of cerebral activation. Human brain mapping, 4(1), 58-73.

[24] Nieto-Castanon, A. (2020). Cluster-level inferences. In Handbook of functional connectivity Magnetic Resonance Imaging methods in CONN (pp. 83–104). Hilbert Press.

[25] Chumbley, J., Worsley, K., Flandin, G., & Friston, K. (2010). Topological FDR for neuroimaging. Neuroimage, 49(4), 3057-3064.
